# Silicon reduces the iron uptake in rice and induces iron homeostasis related genes

**DOI:** 10.1038/s41598-020-61718-4

**Published:** 2020-03-19

**Authors:** Martin Becker, Ngoc Sang Ngo, Manfred Karl Adolf Schenk

**Affiliations:** 1 Institute of Plant Nutrition, Faculty of Natural Sciences, Leibniz University Hannover, Herrenhäuser Str. 2, 30419 Hannover, Germany; 2 Present Address: Leibniz Institute of Plant Genetics and Crop Plant Research (IPK), Plant Reproductive Biology; Corrensstr. 3; D-06466 Seeland/OT, Gatersleben, Germany

**Keywords:** Plant physiology, Plant signalling

## Abstract

Gramineous plants take up silicon (Si) that enhances the formation of exodermal Casparian bands (CBs) in the roots of rice (*Oryza sativa* L.). Furthermore, it is known that Si supply reduces the concentration of Fe in rice shoots. We hypothesized that the Si-enhanced CB formation in the exodermis reduces in the flux of Fe in the apoplast and the uptake of Fe loaded deoxymugineic acid. Thus, the effect of silicic acid supply at varied Fe concentrations and Fe forms was investigated in nutrient solution. The Fe concentrations in the shoot and apoplastic Fe concentrations in the root were determined and an Affymetrix GeneChip experiment was carried out together with qRT-PCR measurements for observation of transcriptomic reactions. Additionally, the Fe uptake of an overexpression mutant of Os*ABCG*2*5* with an enhanced exodermal CB formation was investigated. The application of silicic acid reduced the Fe concentrations in shoot DM independently of the supplied Fe concentration and Fe form. As a reaction to the Fe shortage, the full cascade of Fe-homeostasis-related genes in the roots was upregulated. Silicic acid supply also decreased the apoplastic Fe concentrations in roots. In addition, an overexpression mutant of Os*ABCG25* with an enhanced CB formation showed a reduced uptake of Fe in excess Fe conditions. The results suggest that the Si-induced CB formation in the exodermis hampers the flux of Fe into the apoplast of the cortex and, thus, Fe uptake of rice grown in nutrient solution which is reflected in the upregulation of Fe homeostasis-related genes.

## Introduction

Silicon (Si) is the second most abundant element in the soil crust and many plants can take up and accumulate Si^1–3^ which has beneficial effects, such as a higher resistance to herbivores or powdery mildew, a higher drought resistance and a higher stability of the leaves^4^. Another effect of Si is the earlier formation of the exodermal Casparian bands (CBs) which appear in submerged grown rice^5,6^. The mechanism is not yet clear^7^ but it is suggested that Si(OH)_4_ forms crosslinks with phenols within cell walls leading to an enhanced CB development. The CBs are depositions of suberin and lignin in anticlinal cell walls of the endodermis of all higher plants and of the exodermis of many plants^8,9^. Endodermal CBs control the unselective bypass flow of water and ions into the vascular bundle^10^. Exodermal CBs are described in rice as controlling radial oxygen loss and water flux^11,12^. It is also indicated in the literature that the exodermal CBs could affect in nutrient acquisition. Due to the late occurrence of CBs during root development and the disruptions of the exodermis from lateral roots, it is mentioned that the effect of exodermal CBs in ion exchange should not be overestimated^13,14^. Accordingly, the flux of apoplastic tracer, such as trisodium 3-hydroxy-5,8,10-pyrene trisulfonat or cis-absicid acid was not hampered by the exodermal CBs^10,15^. But, the exodermal CBs are also described as a diffusion barrier reducing the flux of ions into the cortex^16–19^ and to have a function in controlled substance exchange^20–22^, while positive effects of the exodermal CBs for water management are also described^8^. Furthermore, it has been shown that Si supply reduces the bypass flow of sodium and, thus, the sodium uptake declines^17,18,23,24^.

Rice has exodermal CBs which have a specific function regarding the aerenchyma, which is a root tissue developing from collapsing cortical cells to ensure the oxygen supply of the root^25,26^. Exodermal CBs block the radial oxygen loss in adventitious roots^6^. Previously it was shown that Si enhances the formation of exodermal CBs in adventitious roots of rice and clearly reduces the radial oxygen loss^6,27^. The exodermal CB formation starts with/ without Si application 4–5/8–10 cm from the root tip in the anticlinal cell walls and is completed at 7–8/12–13 cm distance from the root tip, respectively^6,28^.

Silicon also affects nutrient uptake in rice. Previous studies^27,29^ reported that Si supply reduced the Fe uptake of plants grown in nutrient solution and under Fe excess the toxicity symptom leaf bronzing clearly decreased^30^ but causal relationships are still not clear. Iron acquisition in plant kingdom follows two strategies^31^. In dicotyledonous and monocotyledonous non-graminaceous species (strategy I) Fe(III) precipitates are dissolved in the rhizosphere by release of protons and excreted phenols such as coumarins chelate Fe(III)^32^. This complex diffuses to the root surface where the membrane bound ferric reductase reduces Fe(III) to Fe(II) which is taken up by iron related transporter (IRT). The activity of the ferric reductase is enhanced by low pH. Graminaceous plants such as rice follow strategy II in Fe uptake^33^. In strategy II, plants secrete via TOM1 phytosiderophores, such as deoxymugineic acid (DMA), into the rhizosphere that bind to hardly available Fe^III+^. This complex is taken up by yellow stripe-like (YSL) transporters at the root epidermis and, most probably, in the root cortex^34–36^. Under Fe deficiency iron homeostasis genes are regulated and the expression of genes involved in the synthesis of DMA from methionine such as nicotianamine synthase (NAS), nicotianamine aminotransferase (NAAT), and deoxymugineic acid synthase (DMAS) is enhanced. Also genes encoding for mugineic efflux transporters (TOM) and influx transporters of Fe chelates (YSL) are upregulated^37–41^. The rhizosphere is different in plants grown in nutrient solution to those grown in soil since the whole pot volume can be defined as rhizosphere, because of the high diffusion coefficient in water. Thus, DMA is extremely diluted and less effective for Fe acquisition. In nutrient solution, the binding to DMA and the subsequent uptake is discussed to take place in the apoplastic space of the cortex^42^.

The aim of this work is to identify the reasons for the lower Fe content in the leaves of plants grown with Si. We hypothesize that the earlier CB formation in the exodermis reduces the amount of Fe in the apoplast and, thus, reduces the uptake of DMA-loaded Fe.

## Results

### Silicic acid induced differential gene expression

**Figure 1:**
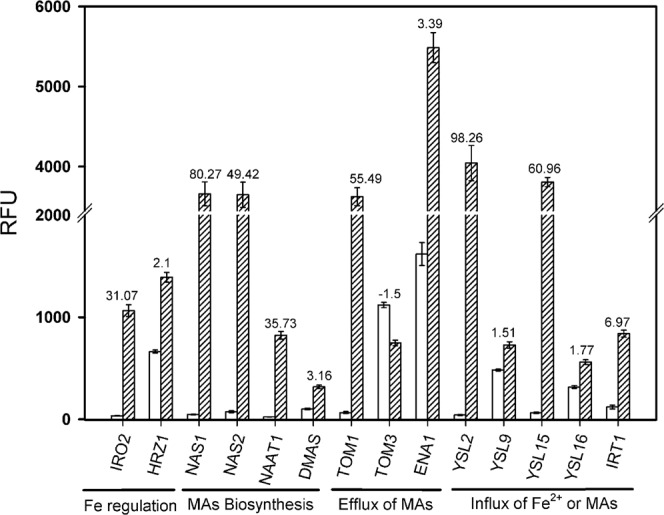
Regulation of Fe homeostasis-related genes through silicic acid (Si) in rice roots from the Affymetrix GeneChip experiment. Yellow dotted bars represent the Si-treated plants grown with Si (30 mg L^−1^) in nutrient solution; blue bars represent plants grow. n with low Si concentrations (3 mg L^−1^). The relative fluorescence unit (RFU) is shown; bars indicate the standard error (SE). Numbers above the bars represent the foldchange (FC). Abbreviations of the genes are: DMAS: Deoxymugineic acid synthase; ENA1: Efflux of nicotianamine 1; HRZ1: Haemerythrin motif-containing RING-& Zinc finger protein 1; IRO2: Iron-related transcription factor 2; IRT1: Iron related transporter 1; NAAT1: Nicotianamine aminotransferase 1; NAS1: Nicotianamine synthase 1; NAS2: Nicotianamine synthase 2; TOM1: Transporter of mugineic acid 1; TOM3: Transporter of mugineic acid 3; and YSL2/9/15/16: yellow stripe like (YSL) transporter 2/9/15/16.

Rice plants were grown with high (+Si, 30 mg L^−1^) or low Si (−Si, 3 mg L^−1^) in nutrient solution for 28 d. The zone 2–6 cm from the tip of adventitious roots was harvested, since here CB development in the exodermis is clearly enhanced by Si supply^28^, and an Affymetrix GeneChip was used to identify differentially regulated genes. We observed 172 upregulated and 207 downregulated genes due to Si treatment with a minimal fold change of 2 at a p-value cut-off of 0.01 (Supplementary Fig. S1). The complete GeneChip experiment can be found under GSE111019 (Gene Expression Omnibus). A list of the 50 most up- and downregulated Si-related genes is given in Supplementary Table S1a,b. Fourteen of the upregulated genes were related to the Fe homeostasis in rice (Fig. 1) such as the Fe-regulating transcription factors Os*IRO2* (LOC_Os01g72370.1) and Os*HRZ1* (LOC_Os01g49470.2).

Additionally, genes for the desoxymugineic acid (DMA) biosynthesis, such as nicotianamine synthase 1/2 (Os*NAS1*, LOC_Os03g19427.1; Os*NAS2* LOC_Os03g19420.2), nicotianamine aminotransferase 1 (Os*NAAT1*, LOC_Os02g20360.1) and deoxymugineic acid synthase (Os*DMAS*, LOC_Os03g13390.2) were upregulated while OsNAS3 was not regulated differentially. The mugineic acid (MA) efflux transporter Os*TOM1* (LOC_Os11g04020.1) was similarly upregulated, while the expression level of Os*TOM3* was decreased. The nicotianamine efflux transporter Os*ENA1* responsible for Fe-MA mobilization in the vacuole was also upregulated. An increased expression level was furthermore observed for genes related to the influx of Fe-MA, such as Os*YSL2* (LOC_Os02g43370.2), Os*YSL9* (LOC_Os04g45860.1), Os*YSL15* (LOC_Os02g43410.2) and Os*YSL16* (LOC_Os04g45900.1). The Fe^2+^ transporter 1 (Os*IRT1*) was also upregulated. A differential regulation of genes involved in Fe-MA storage and sequestration was not observed (Os*Vit1.1/1.2*, LOC_Os04g38940.1/LOC_Os09g23300.1; Os*Fer1/2*, LOC_Os11g01530.1/LOC_Os12g01530.1).

### Fe nutrition as affected by silicic acid at varied Fe supply

**Figure 2:**
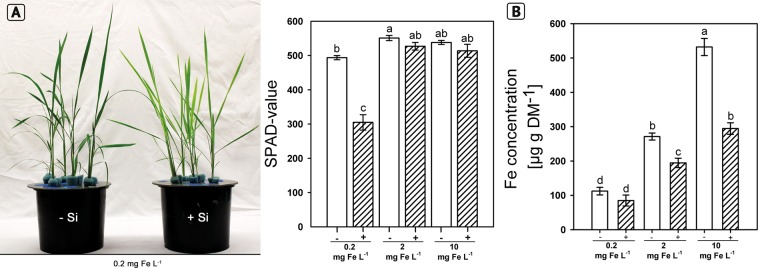
Iron nutritional status of rice plants grown in nutrient solution as affected by silicic acid supply (+Si: 30 mg L^−1^, −Si: 3 mg L^−1^) and Fe supply (Fe^EDDHA^) **(A)** phenotypic differences between rice plants grown in low Fe conditions (0.2 mg L^−1^) and chlorophyll concentration of leaves shown as SPAD value of plants grown with low Fe (0.2 mg L^−1^), optimal Fe (2 mg L^−1^) and high Fe (10 mg L^−1^). **(B)** Iron concentrations in the shoot dry matter (DM). Ruled bars represent the +Si plants; white bars represent plants grown with −Si. Bars indicate the SE. Different letters indicate significant differences between treatments; Bonferroni test with p < 0.05.

The observations suggested that Si supply induced a Fe shortage in rice plants. Thus, we investigated the Fe uptake and the gene expression as affected by Si nutrition at different Fe levels and Fe forms. Plants grown for 28 d in +Si at the lowest Fe supply showed Fe deficiency symptoms (Fig. 2A) compared to the other treatments, which was also reflected in the SPAD values (Fig. 2A). Furthermore, the Fe concentration in shoot DM was reduced by Si supply in all Fe levels and reduction varied between 25 and 32.4% at low and high Fe levels, respectively (Fig. 2B). The Fe concentration in shoot DM was in the 2 mg Fe L^−1^ treatment in the range as reported for well supplied plants^31,43^.

This Si(OH)_4_ effect was not due to an interaction with iron in the nutrient solution since hydrodynamic diameter was not affected as determined by Zeta-potential measurement combined with phase-analysis light scattering technique (Supplementary Fig. S2).

**Figure 3:**
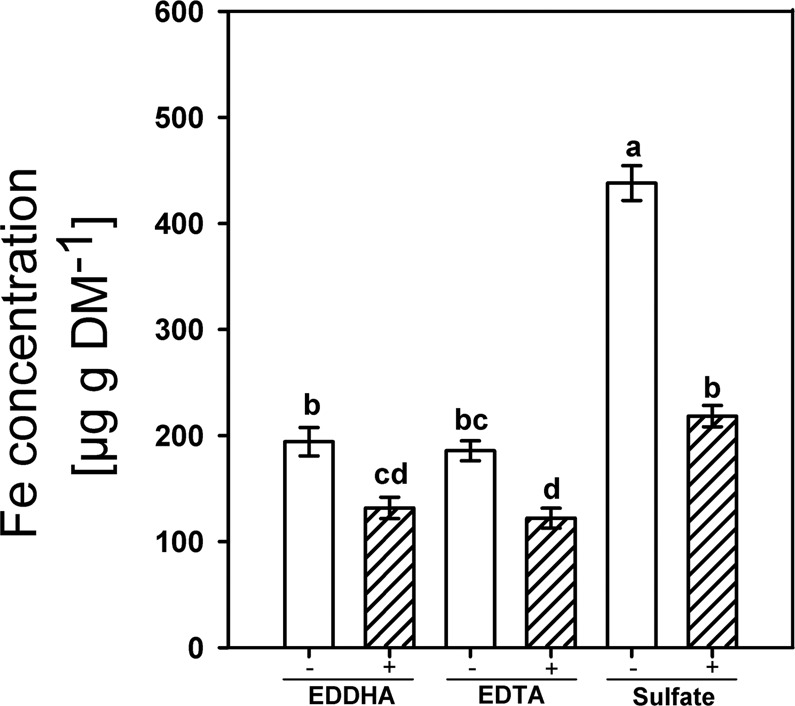
The Fe concentration in the shoot DM of rice as affected by the offered Fe form (2 mg L^−1^) and silicic acid supply (+Si: 30 mg L^−1^, −Si: 3 mg L^−1^) in nutrient solution. Ruled bars represent the +Si-treatments; white bars represent −Si treatments. Bars indicate the SE. Different letters indicate significant differences between treatments; Bonferroni test with p < 0.05.

Silicic acid supply also reduced the Fe concentrations in shoot DM independently of the Fe form, as can be seen in Fig. 3. The Si effect was much stronger for plants grown with Fe^(II)^SO_4_ than with Fe^EDDHA^ and Fe^EDTA^. The Fe supply in the form of Fe^(II)^SO_4_ enhanced the Fe concentration in shoot DM by a factor of two compared to the Fe chelates.

There was no difference in the chlorophyll concentrations (SPAD) at harvest between treatments (Supplementary Fig. 3), but plantlets grown in +Si with Fe^EDTA^ or Fe^(II)^SO_4_ showed chlorotic leaves at the beginning of the experiment. This effect was not measurable with the SPAD meter due to the small leaf area of the plantlets.

**Figure 4:**
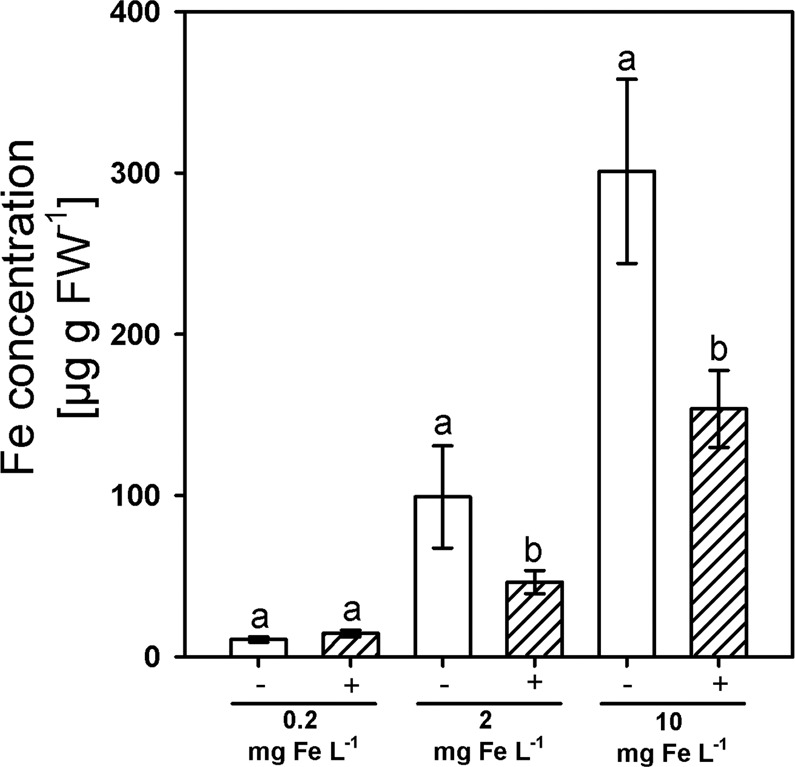
The Fe concentration in the apoplast of adventitious rice roots as affected by Fe supply (Fe^EDDHA^) and silicic acid supply (+Si: 30 mg L^−1^, −Si: 3 mg L^−1^). Ruled bars represent +Si plants; white bars represent −Si plants. Bars indicate the SE. Different letters indicate significant differences between respective treatments; Wilcoxon rank sum test with p < 0.05.

Since silicic acid supply reduced the Fe concentration in shoot DM regardless of the level and the form of Fe supply, we assumed a common mechanism for this Si effect. We hypothesized that the Si-enhanced CB development in the exodermis^6,27^ reduced the Fe flux into the root apoplast where Fe is thought to be bound by DMA and then taken up by YSL transporters. Therefore, the apoplastic Fe concentrations were determined (Fig. 4). They clearly increased with increasing Fe supply and were reduced by Si supply at the two higher Fe supply levels.

**Figure 5:**
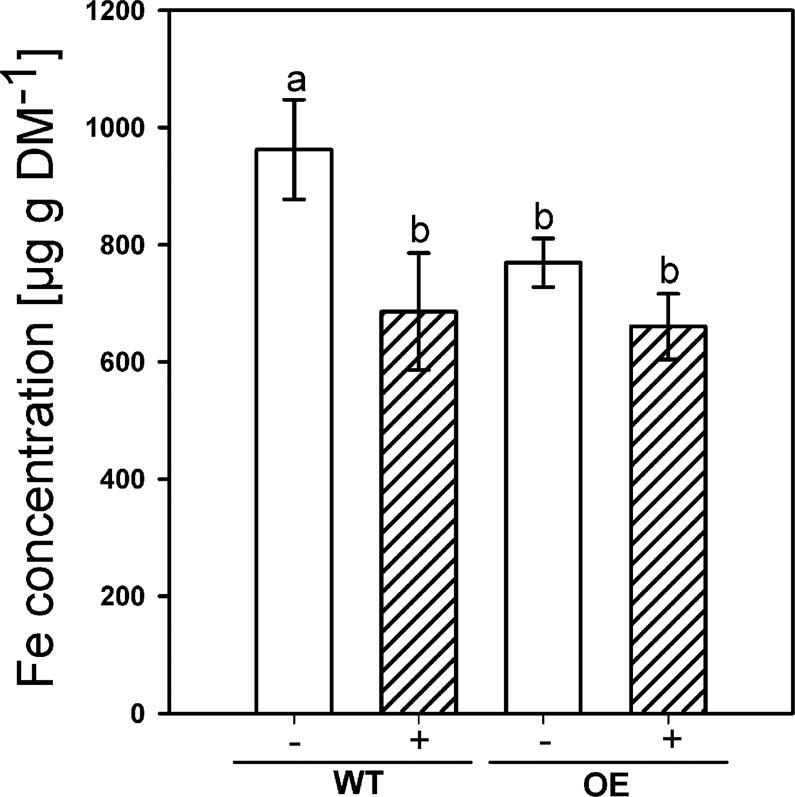
The Fe concentration in the shoot of WT and the overexpression mutant of OsABCG25 grown with excessive amounts of Fe (250 mg L^−1^ FeSO_4_) in nutrient solution. Ruled bars represent the Si-treated plants (+Si, 30 mg L^−1^); white bars represent plants grown with low Si (−Si, 3 mg L^−1^) concentration. Bars indicate the SE. Different letters indicate significant differences between WT plants grown without Si (−WT) and other treatments; students t-test with p < 0.05.

Additionally, an overexpression mutant of the gene Os*ABCG25* (LOC_Os10g30610.1) having an enhanced CB development in the exodermis was used and grown under excessive Fe supply to reinforce the hypothesis^27^. The overexpression mutant grown without Si contained clearly less Fe in shoot DM compared to WT (Fig. 5). The application of Si to WT plants affected the Fe concentration similarly, but the effect was only slightly visible in the mutant.

### Gene expression as affected by silicic acid at varied Fe supply

**Figure 6:**
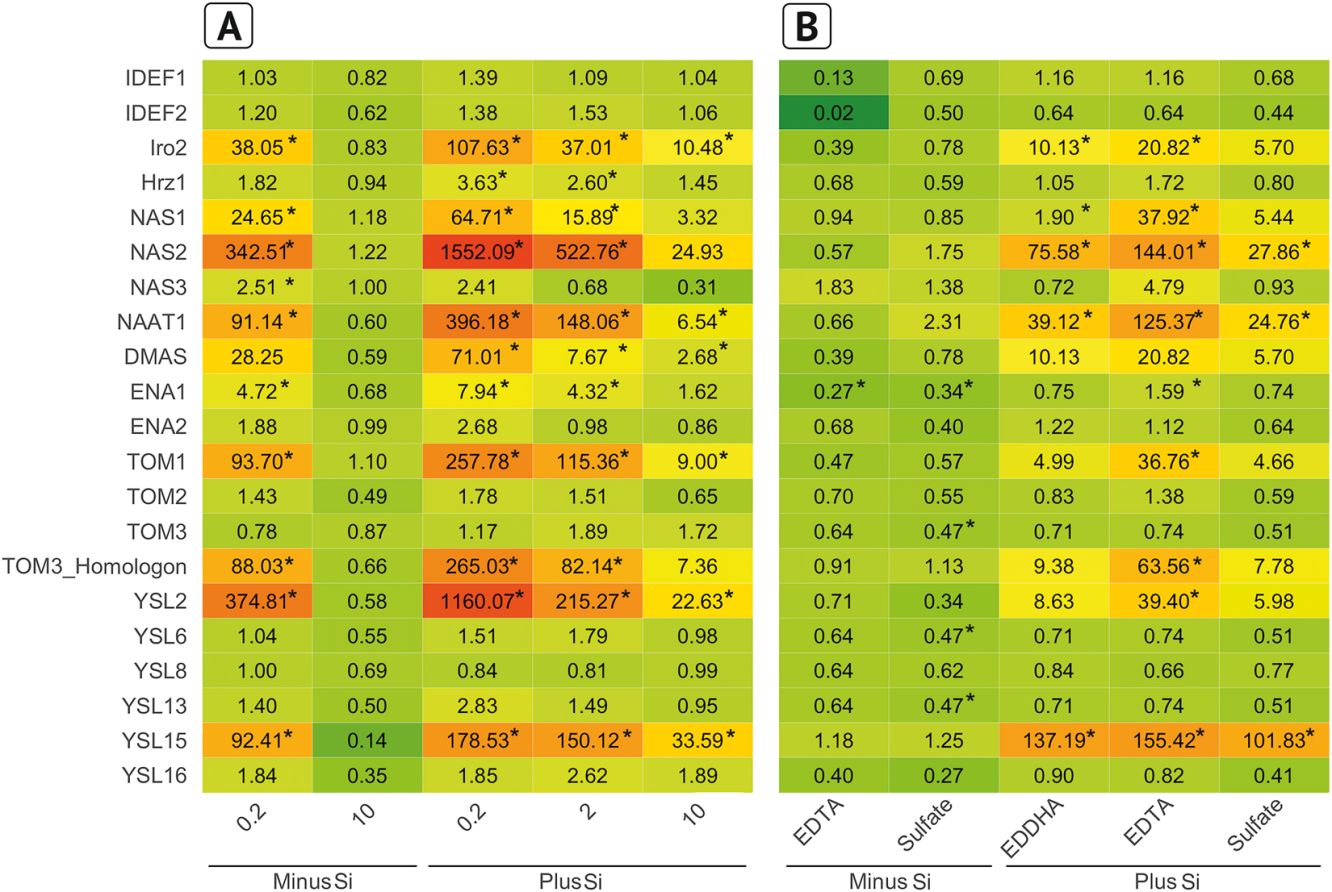
Regulation of Fe homeostasis-related genes in rice roots as affected by Fe concentration and Fe form grown without Si and with Si (3 and 30 mg Si L^−1^, respectively) in nutrient solution. The 2^−∆∆Ct^ values against the control are shown. (**A**) Gene expression in roots of rice plants grown with Fe^EDDHA^ in various concentrations (control = −Si, 2 mg L^−1^ Fe). (**B**) Gene expression in roots of rice plants grown with 2 mg Fe in the form of Fe^EDDHA^, Fe^EDTA^ and Fe^(II)^SO_4_ (control = −Si, Fe^EDDHA^). A star indicates significant differences against the control. Green color indicates downregulation and a red color upregulation compared to the WT grown without Si at optimal Fe conditions (2 mg L^−1^ Fe^EDDHA^).

Silicic acid supply hampered the Fe nutrition of rice and this was reflected in the expression of Fe homeostasis genes. About half of the genes investigated were upregulated at the lowest Fe level without Si supply (Fig. 6A), but not differentially expressed at the highest Fe level compared to the optimal level of 2 mg L^−1^. The expression of the same genes which were upregulated in the treatment 0.2 mg Fe L^−1^ were also enhanced in all Fe levels by Si supply and the expression declined with increasing Fe concentration in the nutrient solution: The transcription factors Os*IRO2* and Os*HRZ1*, the chelate synthesis genes Os*NAS1*, Os*NAS2*, Os*NAAT1* and Os*DMAS*, the DMA efflux transporter genes Os*TOM1* and the Os*TOM3* homologon (*Os12g0132500*), and finally the Fe-MA uptake transporters YSL2 and YSL15. The expression of Os*ENA1* coding for a Fe-DMA/nicotianamine (NA) transporter from the vacuole was also enhanced.

A similar pattern of Si-induced gene expression was observed in the experiment with different Fe forms (Fig. 6B).

## Discussion

Silicon reduced the concentration of Fe in the shoot of plants independently of the supplied Fe concentration and Fe form and triggered Fe deficiency symptoms (Figs. 2, 3). This agrees well with previous reports that Si supply decreases the Fe concentration in shoots of rice grown in nutrient solution^27^. Si supply also reduced the Fe concentration in shoot DM of rice grown in nutrient solution under Fe excess nutrition^44^. We further observed a reduction of the Fe concentration in the root apoplast (Fig. 4). Thus, we suggest that the Si-enhanced formation of the exodermal CBs led to a decrease of the Fe concentration in the apoplast and reduced Fe uptake.

The formation of exodermal CBs is promoted by Si and CBs occur earlier in the adventitious roots of rice^6^ and in the roots of *Zea mays*, *Allium cepa*, *Tradescantia virginiana* and *Guizotia abyssinica*^28^. The endodermal CBs are well described to be an effective barrier for unselective bypass flow of substances from the apoplastic space into the vascular bundle^13,20,45,46^ and the exodermal CBs were shown to hamper the flux of oxygen from the aerenchyma into the rhizosphere of rice roots^5,6,47–49^. Furthermore, it was observed that Si reduces sodium uptake in rice due to a reduced transpirational bypass flow^17,18^. However, the impact of exodermal CBs in nutrient and water uptake is controversially discussed in the literature. Apoplastic tracers are shown to be permeable in roots with an exodermis, such as trisodium 3-hydroxy-5,8,10-PTS or *cis*-ABA^10,15^. However, it is discussed that the lateral root formation may interrupt the exodermal CBs, resulting in a reduced barrier function of the exodermis^8,50^. Earlier results from Hinrichs *et al*.^27^ suggested the exodermal CBs as a diffusion barrier for Fe into the apoplastic space. We observed that Si supply reduced the concentration of apoplastic Fe (Fig. 4), supporting the evidence that CBs act as a barrier for the flux of Fe into the root cortex. The apoplastic Fe concentration was in the range of 46 up to 99 µg g FW^−1^ (1–2 µmol g FW^−1^) at optimal Fe conditions, as observed in other studies^51^. Furthermore, a mutant overexpressing the ABC transporter Os*ABCG25* resulting in enhanced CB development^27^ also had a decreased concentration of Fe in the shoot DM (Fig. 5). The presented results support the hypothesis that the exodermal CBs hamper the flux of Fe from the nutrient solution into the apoplast of the root cortex.

Rice is a strategy II plant^52^, releasing DMA by transporters into the rhizosphere of plants grown in soil where DMA binds to Fe ions. The Fe-loaded DMA is taken up into the symplast by transporters located in the root epidermis and in the cortex. Regarding plants grown in nutrient solution, however, the whole solution volume can be considered as rhizosphere because of the large diffusion coefficient and the DMA secreted will be extremely diluted and less effective for Fe acquisition. Thus, it was further suggested that under these conditions, the uptake of Fe most probably takes place from the apoplast where the excreted DMA binds to Fe and then the complex is taken up into the cortex cells^42^.

**Figure 7:**
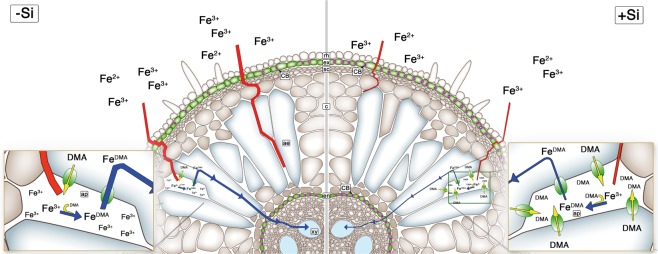
The Fe acquisition in rice roots grown in nutrient solution as affected by Si supply. Without Si supply, the Fe flux into the apoplast of the cortex is not severely restricted by CB in the exodermis. In the apoplast of the cortex Fe binds to excreted DMA and is taken up into the cortex cells and passes the endodermis symplastically into the xylem. Silicon supply induces the CB formation in the exodermis and hampers the flux of Fe into the apoplastic space where less Fe can be bound to DMA resulting in a reduced symplastic flux into the xylem. Abbreviations are rh: rhizodermis, ex: exodermis, sc: sclerenchyma, CB: Casparian band, c: cortex, ae: aerenchyma, ap: apoplast, en: endodermis, xy: xylem. Red and blue lines indicate apoplastic and symplastic flux, respectively.

A scheme displaying the suggested functions of Si in Fe acquisition of plants grown in nutrient solution is presented in Fig. 7. The Si-induced CB formation in the exodermis hampers the flow of Fe into the apoplastic space where less Fe can be bound to DMA and taken up into the symplast. In agreement with this model, Si supply induced upregulation of Fe homeostasis genes. This proposed mechanism of Si function is in line with the apoplastic obstructuion hypothesis as working model to understand the role of Si in plant biology^7^. One premise of this model is that there is no biochemical role for Si(OH)_4_ in terms of interactions with enzymes or other intracellular constituents but that such reactions are based on indirect effects caused by Si deposition and reactions in the apoplast. In a certain way this agrees with a lack of specifity of Si-transporters which originate according to the horizontal gene transfer model from bacterial As-transporters^2^.

**Figure 8:**
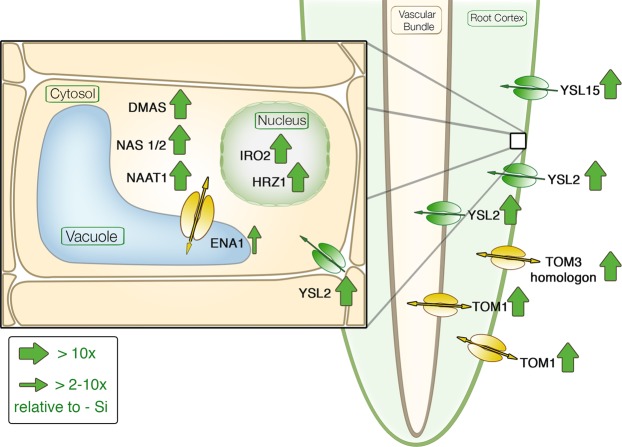
Schematic figure of Si-induced Fe homeostasis-related genes in rice displaying the function of significantly regulated genes from Fig. 6. A big arrow indicates an upregulation compared to the control (−Si; plants grown without Si) with a minimum of tenfold; a small arrow indicates an upregulation with a minimum of twofold. Please see Fig. 1 for abbreviations.

The Si-induced reduction of Fe uptake was also reflected in gene expression. The transcriptomic data displayed the full range of Fe deficiency-induced reaction cascade in rice (Figs. 1 and 6). An overview of the interacting genes and their activity sites is given in Fig. 8. The reaction cascade starts with the transcription factors Os*IDEF1* or 2, which both are regulating elements of Os*TOM1*, Os*IRO*2, Os*NAS1* +*2*, Os*NAAT1* and Os*DMAS*. But not OsNAS3 which is only active under excess Fe conditions^53^. The transcript level of Os*IDEF1* +2 is not influenced by Fe availability, but only the protein binding to the DNA is enhanced during Fe deficiency^40,54^. This agrees well with our results that Si did not affect their expression levels (Fig. 6). The activity of Os*HRZ1* is enhanced by IDEF1 and is suggested as negative feedback loop^55^. Putatively, Os*IRO2* activates the biosynthesis of NA and, thus, the synthesis of DMA and MA in subcellular organelles through Os*NAS1*, Os*NAS2*, Os*NAAT1* and Os*DMAS*^39^. The DMA is then transported via OsTOM1 into the apoplast^30,40^.

TOM3 was not affected by Si in rice roots, while the TOM3 homologon (*Os12g0*13*2500*) was highly upregulated, which agrees with the literature^35^. An upregulation of the TOM3 homologon was found, but not of Os*TOM3* in the epidermis of rice roots in Fe-deficient conditions.

The putative vacuole transporter Os*ENA1*^40^ transporting NA was upregulated. Os*ENA1* is thought to mobilize Fe during Fe shortage from the vacuole. Further characterization of Os*ENA1* is necessary, since the recent research is only based on *in vitro* motif predictions. Finally, Fe-loaded DMA is taken up by different YSL transporters. Os*YSL2* is described as expressed mainly in phloem companion cells for the transport of Fe^II^-NA^34^. Because of the Si-induced shortage of Fe, Os*YSL2* was highly upregulated. Most probably, Os*YSL2* is responsible for the uptake and distribution of Fe^II^-NA by Si-induced Fe shortage. No regulation was observed for Os*YSL9* and Os*YSL13*. Os*YSL9* was recently shown to be expressed in the cortical cells of roots^36^ and Os*YSL13* was described in cortex and epidermis cells^35^. Both, Os*YSL9* and *13* transporters do not seem to be involved in the Si-dependent reaction. Os*YSL15* was highly upregulated, but not Os*YSL16*; both are located in the exodermis or in the cortex. Os*YSL15*, transporting Fe^III^DMA, is under Fe deficiency induced in all root parts^56^.

Beneficial effects of Si supply on Fe stress alleviation in barley are reported by Nikolic *et al*.^56^. On the first glance, this seems to be in contrast to our results. However we investigated the Fe uptake from nutrient solution whereas Nikolic *et al*. studied the mobilization of Fe in plants after transfer to -Fe conditions.

## Conclusion

Silicic acid supply in nutrient solution stimulates the formation of exodermal CBs. It is suggested that this barrier reduced the flux of Fe into the apoplastic space where less Fe was available for chelation by DMA and plant uptake. The plant reacted with an upregulation of Fe homeostasis-related genes and a higher production of Fe-chelating substances as a metabolic answer to the Fe shortage. This response was not sufficient to overcome the Si effect, since shoot Fe concentration was still decreased. This Si role might be specific for rice grown in nutrient solution, because binding of Fe to DMA is discussed to occur in the root apoplast since the excreted DMA is extremely diluted in the nutrient solution compared to the situation soil.

## Material and methods

### Plant material, growth conditions and harvest

Seeds of *Oryza sativa ssp. Japonica* cv. Selenio were first surface sterilized in 70% ethanol for 1 min and then for 30 min in 3.5% NaClO, followed by washing in sterile water three times. Germination was carried out for several days between two layers of filter paper standing in sterile tap water for 14 d. Seedlings were transferred to non-aerated nutrient solution in 5-L pots containing 0.43 mM NH_4_NO_3_, 0.32 mM NaH_2_PO_4_ × 2 × H_2_O, 0.51 mM K_2_SO_4_, 1 mM Ca(NO_3_)_2_ × 4 × H_2_O, 1.6 mM MgSO_4_ × 7 × H_2_O, 1.82 µM MnSO_4_ × H_2_O, 0.03 µM (NH_4_)_6_Mo_7_O_24_ × 4 × H_2_O, 9 µM H_3_BO_3_, 0.6 µM ZnSO_4_ × 7 × H_2_O and 0.15 µM CuSO_4_ × 5 × H_2_O. Iron was added as Sequestren™ (Fe^EDDHA^, Syngenta Agro GmbH, Maintal, Germany) for Fe gradient experiment in three different levels, low Fe (3.58 µM Fe^EDDHA^), optimal Fe (35.81 µM Fe^EDDHA^) and high Fe (179.05 µM Fe^EDDHA^) corresponding to 0.2, 2 and10 mg Fe, respectively, in nutrient solution. An amount of 2 mg Fe L^−1^ was added as Fe^EDDHA^, Fetrilon™ (Fe^EDTA^, BASF, Ludwigshafen, Germany) and iron sulfate (Fe^II^SO_4_ × 7 × H_2_O, CAS: 7782-63-0, KMF, Lohmar, Germany) for the Fe form experiments. Plants were cultivated for three weeks as in the Fe gradient experiment, and the last week with 4.475 mM Fe supplied as Fe^II^SO_4_ × 7 × H_2_O for the growth of the mutants of Os*ABCG25*. Please see Hinrichs *et al*.^27^ for characterization of Os*ABCG25*. The Si was applied as silica gel (SiO_2_, Carl Roth, Karslruhe, Germany). A stock solution was prepared by addition of 5 kg silica gel in 1000 L H_2_O^dest.^ at RT in the dark. The silica gel dissolved slowly until a Si-concentration of 50 mg*l^−1^ was reached. This stock solution was diluted with H_2_O^dest.^ to a working-solution of 30 mg*l^−1^ according to Fleck *et al*.^6^. The H_2_O^dest.^ had a basal Si-concentration of 3 mg*l^−1^ due to technical reasons. The resulting Si concentrations in the plant are in the range of 0.1 and 60 mg g^−1^ shoot DM according to previous findings^57^. The pH value of the nutrient solution was adjusted to 6.0 by the addition of 10% H_2_SO_4_ or 5 M KOH. The nutrient solution was renewed weekly for the first two weeks, after two weeks, and subsequently the nutrient solution was changed twice a week until harvest. Plants were cultivated in a climate chamber (photoperiod 16/8 h light/dark; temperature, 25/20 °C day/night; 75% relative humidity and a light intensity of 220 µmol m^−2^ s^−1^) for 28 d in nutrient solution.

After 28 d in nutrient solution, the root zones 4–6 cm from the the root tip were harvested, transferred immediately to liquid nitrogen and stored at −80 °C for transcript analysis. Only adventitious roots were harvested for Fe determination in the apoplast. Shoot and root were separated, dried at 60 °C for 4 d and ground for element analysis. The content of chlorophyll was measured spectrometrically using an N-Tester (Yara, Dülmen, Germany).

### Fe determination apoplast

Adventitious roots were separated and Fe on the root surface was removed through rinsing in anaerobe washing solution (12 mM Na_2_S_2_O_4_, 10 mM MES, 0.5 mM Ca(NO_3_)_2_, pH = 5.5) for 5 s. Subsequently, the roots were washed three times in H_2_O and transferred into 21 ml anaerobe reaction solution (1.5 mM 2.2-Bipyridyl, 10 mM MES, 0.5 mM Ca(NO_3_)_2_, pH = 5.5)^51^. The reaction solution with the samples was deoxygenized by N_2_ bubbling in an Erlenmeyer flask covered with a cotton plug for 2 min. After 2 min, 1 ml of a cooled 250 mM Na_2_S_2_O_4_-solution was added using a syringe and incubated by gently shaking for 5 min at room temperature. After incubation, the roots were removed, and the reaction was stopped. The washing solution and reaction solution were stored on ice with constant N_2_-bubbling until usage. The Fe was determined by measuring the A520 of the filtrated reaction solution.

### Transcript analysis

Frozen root material was ground under liquid nitrogen and the RNA was isolated using NucleoSpin RNA Plant (Macherey-Nagel, Düren, Germany), following the manufacturer’s instructions. The RNA quality was determined electrophoretically by 1% Agarosegel and fluoretically using a Nanophotometer (Implen, Munich, Germany). The RNA (1 µg), oligo(dT)18-Primer (0.25 µg) and random hexamer primer (0.25 µg) were used to synthesize first-strand cDNA using the Revert Aid™ H Minus First Strand cDNA Synthesis Kit (Fermentas, St. Leon-Rot, Germany), following the manufacturer’s instructions for GC-rich templates.

The RNA was additionally measured by capillary electrophometric by a bioanalyzer (Agilent 2100 bioanalyzer, Agilent Technologies) using a RNA 6000 nano assay in the GeneChip experiment. Only samples with a RNA integrity number over 9.5 were used for the GeneChip experiment. A set of sixteen Genechips were used for the experiment, consisting of two treatments (+Si/−Si), four biological repeats and two technical repeats of the Affymetrix GeneChip experiment. One biological repeat consisted of a pool of the RNA of five plants grown in the same 5-L pot.

An amount of 50 ng cDNA was used as a template in 10 µl reaction mix of the SYBR Premix Ex Taq (Tli RNase H Plus) (Takara Bio Europe SAS, Saint-Germainen-Laye, France) and 0.2 µM forward and reverse primer in the quantitative real time PCR (qRT-PCR) experiments. The qRT-PCR runs were performed in the CFX96 cycler (Bio-Rad, München, Germany), using an initial 95 °C step for 30 s, followed by 40 cycles of 95 °C for 5 s, and 60 °C for 30 s, and a final melting curve procedure with a stepwise increment of 0.5 °C ranging from 65 to 95 °C.

The eukaryotic elongation factor 1-alpha (eF1-α, LOC_Os03g08020.1) and Ubiquitin-40S ribosomal protein S27a-1 (UBQ5, LOC_Os01g22490) were used as an endogenous control due to its stable transcript abundance in rice^58,59^. The geometric mean of the Ct values of the two endogenous control genes was calculated according to the literature^60^, and this was used to calculate the foldover reference of the different samples (2-delta Ct). A list of primer sequences used can be found in Supplementary Table S1. The primer was tested for specificity in a ten times dilution series starting with 50 ng cDNA and then sequencing of the PCR product. Three technical and four biological replicates were used for each target in qRT-PCR. The relative quantity was calculated using the R-Macro “qpcrmix”^61^, based on the 2^−ΔΔCT^ method.

### Chemical analysis

For the determination of the Si concentrations in the shoot and root, 200 mg dried plant matter was digested in 3 ml 65% HNO_3_, 2 ml H2O and 2 ml 30% H_2_O_2_ in a microwave for 12 min at 190 °C, then diluted with 20 ml 10% NaOH, neutralized with HNO_3_^62^ and filled up to a final volume of 100 ml.

In order to determine the Fe, 50 mg of dried and ground shoot matter was digested in 2 ml 65% HNO_3_, 2 ml H_2_O and 0.5 ml 30% H_2_O_2_ in a microwave for 25 min at 190 °C and then diluted with distilled water to 25 ml.

The Si and Fe concentrations in the plant extracts and nutrient solutions- were measured by ICP-MS (7500c Agilent Technologies).

### Zeta potential measurements

The hydrodynamic particle size of possible precipitates of the elements Si and Fe in the nutrient solution was determined by the Zeta potential analyzer with light scattering technique (ZetaPALS, Brookhaven, Holtsville,NY, USA)^63^. Freshly prepared nutrient solution without Fe and with Si and nutrient solution with Fe^EDDHA^ with and without Si were measured immediately or incubated for three and seven days in the same conditions as used for growth of rice in the dark. An aliquot of 1.6 ml was transferred in a cuvette and measurement was performed with ten runs each partitioned in 20 cycles, whereby the mean is represented in the figure.

### Statistical analysis

All treatments were replicated four times unless stated otherwise and the mean of the treatments were compared using t-test, Tukey or Bonferroni test utilizing Sigma Plot (Systat Software Inc., San Jose, USA). The statistical qRT-PCR analysis was performed using the R-Macro of Steibel *et al*.^61^. A students t-test was used with unpaired correlated p-Value for the raw Affymetrix GeneChip data using “GeneSpring” Software (Version 12.0, Agilent). A ranking of candidates was carried out using the “Omics Explorer” (Version 3, Qulcore).

The Affymetrix experiment, including the RNA quality control, GeneChip hybridization, normalization with GeneSpring Software and ranking with the Omics Explorer, was carried out by Dr. Dittrich-Breiholz at the Research Core Unit “Transcriptomics,” Institute of Cell Biochemistry, Hannover Medical School.

## Supplementary information

Supplementary information
