# Supplementary material for: Silicon reduces the iron uptake in rice and induces iron homeostasis related genes
Source: Sci Rep. 2020 Mar 19;10:5079. doi: 10.1038/s41598-020-61718-4 (PMC7081191; doi:10.1038/s41598-020-61718-4)
Supplement: Supplementary file 1 — Supplementary information [file 41598_2020_61718_MOESM1_ESM.pdf]

Silicon reduces the iron uptake in rice and induces iron homeostasis related genes

Martin Becker, Ngoc Sang Ngo, Manfred Karl Adolf Schenk

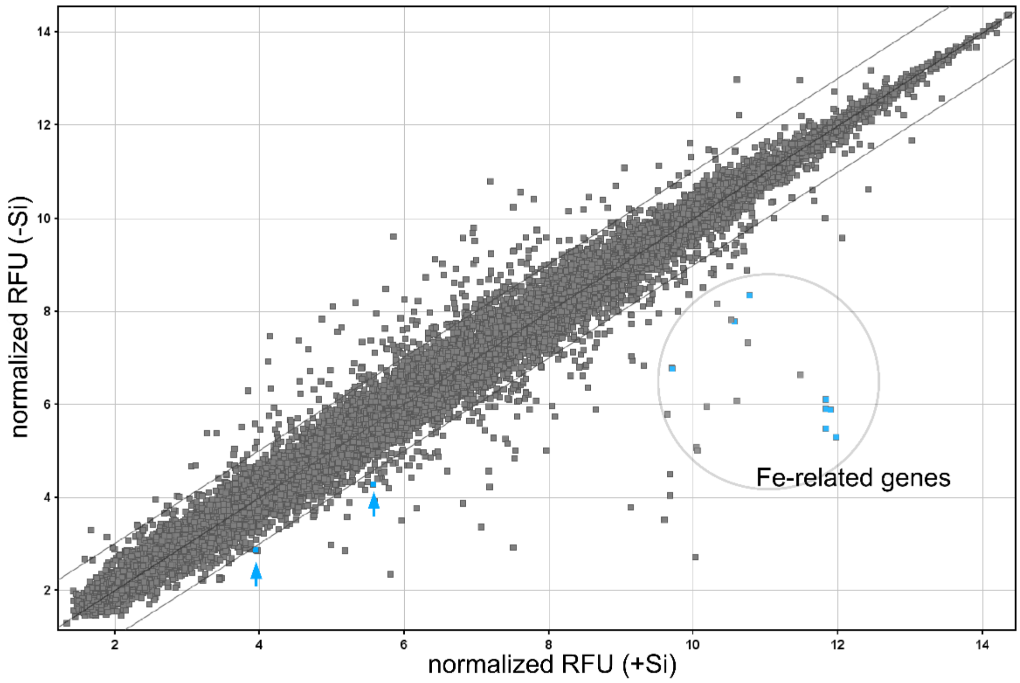

<u>Supplementary Figure S1:</u> Scatterplot of genes up- or downregulated through Si-treatment. The lines indicate the minimum fold change cut-off of 2 in the data, the Fe-related genes are highlighted in blue and with a circle. Data are normalized relative fluorescence units (RFU) from the Affymetrix GeneChip experiment with a p-value cut-off of 0.01. n=8: Multiple Testing Correction: Benjamini-Hochberg.

Supplementary table S1a: Upregulated genes in rice roots due to silicon-treatment. Shown are the 50 most upregulated genes. FC = Foldchange

Identifyer	Gene description	FC
LOC_Os10g11889	expressed protein	155,94
LOC_Os02g43370	OsYSL2	100,91
LOC_Os03g19427	OsNAS1	80,80
LOC_Os03g46454	metal cation transporter, putative, expressed	67,17
LOC_Os02g43410	Iron-phytosiderophore transporter YSL15	63,70
LOC_Os11g04020	OsTOM1	59,90
LOC_Os03g19420	OsNAS2	52,18
LOC_Os12g18410	expressed protein	48,94
LOC_Os03g41926	hypothetical protein	40,05
LOC_Os02g20360	OsNAAT1	35,89
LOC_Os02g38084	hypothetical protein	32,95
LOC_Os01g72370	OsIRO2	31,13
LOC_Os07g15460	OsNRAMP1	28,49
LOC_Os12g32400	helix-loop-helix DNA-binding domain containing protein	23,77
LOC_Os02g02170	OsNRT2.1	22,82
LOC_Os11g15624	expressed protein	18,65
LOC_Os12g38270	OsMT4A	14,38
LOC_Os02g02190	OsNRT2.2	12,82
LOC_Os09g07210	hypothetical protein	10,87
LOC_Os12g38051	OsMT4B	10,61
LOC_Os03g31714	hypothetical protein	7,69
LOC_Os06g38930	LRR EXS 2	7,61
LOC_Os03g46470	OsIRT1	7,60
LOC_Os01g45914	expressed protein	7,60
LOC_Os03g51530	expressed protein	7,39
LOC_Os01g46720	expressed protein	6,79
LOC_Os01g65110	POT family protein, expressed	6,68
LOC_Os03g54000	oligopeptide transporter, putative, expressed	6,44
LOC_Os03g32490	DUF1230 domain containing protein, expressed	6,38
Os01g0810900	protein predicted	6,03
LOC_Os03g41932	hypothetical protein	5,78
LOC_Os03g12510	OsHB2	5,53
LOC_Os03g26210	helix-loop-helix DNA-binding domain containing protein	5,52
LOC_Os03g61490	expressed protein	5,44
LOC_Os02g37260	hypothetical protein	5,35
LOC_Os10g30410	cytochrome P450 71D7, putative, expressed	5,20
LOC_Os06g05020	early nodulin 93 ENOD93 protein, putative, expressed	5,04
LOC_Os06g19095	expressed protein	4,94
LOC_Os09g04360	hypothetical protein	4,79
LOC_Os01g04430	conserved hypothetical protein	4,62
LOC_Os09g39090	vignain precursor, putative, expressed	4,47
LOC_Os11g33950	conserved hypothetical protein	4,46
LOC_Os10g28080	OsCHIB1	4,35
LOC_Os12g13340	expressed protein	4,22
LOC_Os11g45510	expressed protein	4,17
LOC_Os08g25839	expressed protein	4,06
LOC_Os08g25999	hypothetical protein	4,02
LOC_Os06g04990	early nodulin 93 ENOD93 protein, putative, expressed	3,86
LOC_Os02g28130	fasciclin-like arabinogalactan protein 8 precursor, putative	3,61
LOC_Os07g46860	sex determination protein tasselseed-2, putative	3,60

Supplementary table S1b: Downregulated genes in rice roots due to silicon-treatment. Shown are the 50 most downregulated genes. FC = Foldchange

Identifyer	Gene description	FC
LOC_Os03g38950	expressed protein	-2,11
LOC_Os08g37180	patatin, putative, expressed	-2,11
LOC_Os03g45280	dehydrin, putative, expressed	-2,11
LOC_Os09g31478	auxin efflux carrier component, putative, expressed	-2,10
LOC_Os09g21900	expressed protein	-2,09
Os03g0700400	Putative linoleate 9S-lipoxygenase 3 (LOX3_ORYSJ)	-2,08
LOC_Os01g58530	NB-ARC/LRR disease resistance protein, putative, expressed	-2,08
LOC_Os01g66890	BTBZ1 - Bric-a-Brac, Tramtrack,	-2,08
LOC_Os01g60340	NTMC2Type1.1 protein, putative	-2,08
LOC_Os08g10630	OsZIP4	-2,08
LOC_Os11g18730	Beta-D-xylosidase	-2,08
LOC_Os12g43380	Thaumatin-like protein	-2,08
LOC_Os03g53020	Transcription factor BHLH148	-2,07
LOC_Os07g03377	SCP-like extracellular protein	-2,07
LOC_Os12g08730	OsTRXM	-2,07
LOC_Os07g03288	SCP-like extracellular protein	-2,07
LOC_Os02g32580	expressed protein	-2,07
LOC_Os01g73940	expressed protein	-2,07
LOC_Os01g07370	KIP1, putative, expressed	-2,07
LOC_Os01g04050	BBTI12 - Bowman-Birk type bran trypsin inhibitor precursor	-2,06
LOC_Os10g40460	LTPL141 - Protease inhibitor/seed storage/LTP family	-2,06
LOC_Os11g25780	PB1 domain containing protein, expressed	-2,06
LOC_Os07g19000	LTPL41 - Protease inhibitor/seed storage/LTP family protein	-2,06
LOC_Os03g26910	OsTPP9	-2,06
LOC_Os06g41850	SMP-30/Gluconolaconase/LRE-like region containing	-2,06
LOC_Os12g27370	AT hook motif-containing protein, putative	-2,05
LOC_Os03g36534	expressed protein	-2,05
Os01g0892300	protein predicted	-2,04
LOC_Os04g12499	amino acid transporter protein, putative, expressed	-2,04
Os03g0195900	protein predicted	-2,04
LOC_Os01g54340	plant-specific domain TIGR01615 family protein, expressed	-2,04
LOC_Os03g01880	OsLOGL3	-2,04
Os07g0167700	protein predicted	-2,04
LOC_Os03g12790	MATE efflux family protein, putative, expressed	-2,04
LOC_Os01g29804	expressed protein	-2,04
LOC_Os07g03467	SCP-like extracellular protein, expressed	-2,03
LOC_Os11g47510	glycosyl hydrolase, putative, expressed	-2,03
LOC_Os11g32650	OsCHS1	-2,03
LOC_Os10g40640	glycosyl transferase 8 domain containing protein, putative	-2,03
LOC_Os08g09010	OsGER6	-2,02
LOC_Os03g03080	hypothetical protein	-2,02
LOC_Os11g25454	cytokinin-N-glucosyltransferase 1, putative, expressed	-2,02
LOC_Os06g50940	GDSL-like lipase/acylhydrolase, putative, expressed	-2,02
LOC_Os06g47640	OsCML29 - Calmodulin-related calcium sensor protein	-2,01
LOC_Os05g32520	conserved hypothetical protein	-2,01
LOC_Os06g20900	expressed protein	-2,01
LOC_Os02g15200	hypothetical protein	-2,01
LOC_Os07g49360	peroxidase precursor, putative, expressed	-2,00
LOC_Os06g14030	potassium channel SKOR, putative, expressed	-2,00
LOC_Os06g48510	choline monooxygenase, putative	-2,00

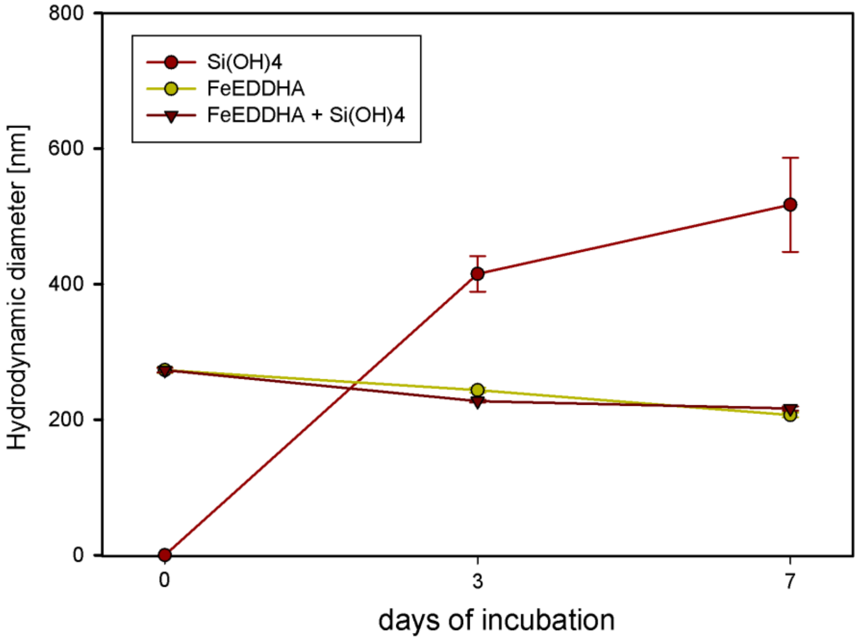

Supplementary Figure S2: Hydrodynamic diameter in standard nutrient solution a) without Fe and with Si supply (Si(OH)4), b) with Fe^{EDDHA} and with Si (FeEDDHA + Si(OH)4), c) with Fe ^{EDDHA} and without Si (FeEDDHA) as affected by duration of incubation. Concentrations were as used in the nutrient solution. Bars indicates the standard-deviation. n=3**.**

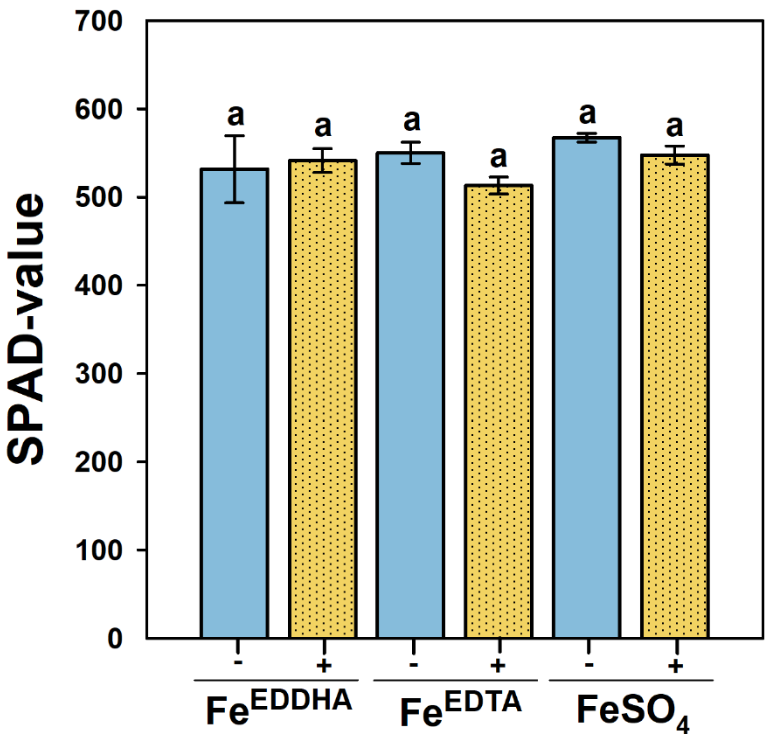

Supplementary Figure S3: Chlorophyll content of the leaves shown as SPAD value as affected by Fe supply in form of EDDHA, EDTA and $Fe^{(II)}SO_4$ grown with (+) and without (-) Si (30 / 3 mg L^{-1}) in nutrient solution. Yellow dotted bars represent the + Si plants; blue bars represent plants grown with -Si. Bars indicate the SE. Different letters indicate significant differences between treatments; Bonferroni-test with $p < 0.05$

Supplementary table S2: List of used primers for qRT-PCR analysis.

Gene assignment	Locus	Orientation	Oligo sequence
Os*DMAS*	LOC_Os03g13390.2	5'-3'<br>5'-3'	GCCGGCATCCCGCAGCGGAAGATCA<br>CTCTCTCTCTCGCACGTGCTAGCGT
Os*ENA1*	LOC_Os11g05390.1	5'-3'<br>5'-3'	TACTTCATTTCAGAACAAGC<br>GCATTTGGCTTCTGGCTTAA
Os*ENA2*	LOC_Os06g48060.1	5'-3'<br>5'-3'	TTCGTCTTCCTCTCGACCTCTC<br>TCCCTGACATCATGCTCTGAAGC
Os*IDEF1*	LOC_Os08g01090	5'-3'<br>5'-3'	ATGGACGACATGGTGCTCC<br>CTAGGGATTTGTTGTCTGCT
Os*IDEF2*	LOC_Os05g35170.1	5'-3'<br>5'-3'	TCTCTGCTGGTTTCTCAAAGGATG<br>GCAGCAAGGGAAACATTTCAGC
Os*IRO2*	LOC_Os01g72370.1	5'-3'<br>5'-3'	CACCAGCACCACCAACTGCAAACCAG<br>GCTTTGTTCCTGACGACTTTCTCC
Os*HRZ1*	LOC_Os01g49470.2	5'-3'<br>5'-3'	GAATTCCACAAATGCCGGGAGAAAGG<br>AGCCAGCAAGGCGTCCAA
Os*NAAT1*	LOC_Os02g20360.1	5'-3'<br>5'-3'	TAAGAGGATAATTGATTTGCTTAC<br>CTGATCATTCCAATCCTAGTACAAT
Os*NAS1*	LOC_Os03g19427.1	5'-3'<br>5'-3'	GTCTAACAGCCGGACGATCGAAAGG<br>TTTCTCACTGTCATACACAGATGGC
Os*NAS2*	LOC_Os03g19420.2	5'-3'<br>5'-3'	TGAGTGCGTGCATAGTAATCCTGGC<br>CAGACGGTCACAAACACCTCTTGC
Os*NAS3*	LOC_Os07g48980.1	5'-3'<br>5'-3'	CGATCGAGTGTTCGACTGATCACC<br>TCCACCGTCATACTCTCTCTCTCG
Os*TOM1*	LOC_Os11g04020.1	5'-3'<br>5'-3'	GCCCAAGAACGCCAAAATGA<br>GGCTTGAAGGTCAACGCAAG
Os*TOM2*	LOC_Os11g04030.1	5'-3'<br>5'-3'	GGCGCAGGTGTTCTATTCTCTTGG<br>ACCGCCAAGAAAGGTTTGAAGGTC
Os*TOM3*	LOC_Os07g01250.1	5'-3'<br>5'-3'	TGGAGGTAATGCAACGTCCC<br>AAAGACGAGAGCACGGACTG
Os*12g0132500*	Os12g0132500	5'-3'<br>5'-3'	GGCGGTGTTCTATGATGGCT<br>ACTCCTTGTAGGGGATGCCT
Os*YSL13*	LOC_Os04g44300.1	5'-3'<br>5'-3'	TGCAACTGCCTTCCTCATAAACGG<br>ACCGAGTTTCTTTACTTGCTTCGC
Os*YSL2*	LOC_Os02g43370.2	5'-3'<br>5'-3'	GAGGGACAACGGTGTCATTGCTGGT<br>TGCAGAAAAGCCCTCGACGCCAAGA
Os*YSL6*	LOC_Os04g32050.1	5'-3'<br>5'-3'	TGTTGCACTGCGCAAGGTAATG<br>TGTCGCAGTTCCACTGGGATAG
Os*YSL8*	LOC_Os02g02460.1	5'-3'<br>5'-3'	TGGGATGCATAATAAGCCCGATGG<br>CATCCCGATGTTGTTCGCCTTG
Os*YSL15*	LOC_Os02g43410.2	5'-3'<br>5'-3'	GGCTATTAGCTTCGCGGGTA<br>CTCCTTGGGCTGTGTGGAAC
Os*YSL16*	LOC_Os04g45900.1	5'-3'<br>5'-3'	GGTGGATTCGGGTCGTTCTT<br>ATGACCAGCACCTTTCGGAG

Housekeeping Genes

eF1-α	LOC_Os03g08020.1	5'-3'<br>5'-3'	TCAAGTTTGCTGAGCTGGTG<br>AAAACGACCAAGAGGAGGGT
UBQ5	LOC_Os01g22490	5'-3'<br>5'-3'	ACCACTTCGACCGCCACTACT<br>ACGCCTAAGCCTGCTGGTT
